# Standard Modifiable Cardiovascular Risk Factors Mediate the Association Between Elevated Hair Cortisol Concentrations and Coronary Artery Disease

**DOI:** 10.3389/fcvm.2021.765000

**Published:** 2022-01-25

**Authors:** Andreas Stomby, Susanna Strömberg, Elvar Theodorsson, Åshild Olsen Faresjö, Mike Jones, Tomas Faresjö

**Affiliations:** ^1^Division of Prevention, Rehabilitation and Community Medicine, Department of Health, Medicine and Caring Sciences, Linköping University, Linköping, Sweden; ^2^Råslätts vårdcentral, Region Jönköping County, Jönköping, Sweden; ^3^Department of Biomedical and Clinical Science, Clinical Chemistry, Linköping University, Linköping, Sweden; ^4^Division of Society and Health/Public Health, Department of Health, Medicine and Caring Sciences, Linköping University, Linköping, Sweden; ^5^Faculty of Medicine Health and Human Sciences, School of Psychological Sciences, Macquarie University, North Ryde, NSW, Australia

**Keywords:** cortisol, cardiovascular risk factors, biological stress, coronary artery disease, path analysis

## Abstract

**Background:**

Increased cortisol exposure is a risk factor for coronary artery disease (CAD). It is not clear to what degree this risk is independent from the standard modifiable risk factors (SMuRFs) dyslipidemia, hypertension, and diabetes.

**Aim:**

To use path analysis to test the direct and indirect association, mediated by SMuRFs, between long-term cortisol levels measured in hair cortisol concentration (HCC) and CAD.

**Methods:**

Hair was sampled from patients admitted with acute myocardial infarction (*n* = 203) and a population-based sample without a diagnosis or symptoms of CAD (*n* = 3,134). The HCC was analyzed using radioimmunoassay and all the participants reported whether they were diagnosed with or treated for diabetes, hypertension, and hyperlipidemia. Path analysis was used to test to what degree the association between logarithmized (ln) HCC and CAD was direct or indirect, mediated by SMuRFs.

**Results:**

Participants with CAD had elevated HCC compared to those without elevated HCC [median interquartile range (IQR): 75.2 (167.1) vs. 23.6 (35.0) pg/mg, *p* < 0.0001]. Higher HCC was associated with diabetes, hypertension, and hyperlipidemia, which, in turn, were associated with CAD. In path models, 80% of the association between ln(HCC) and CAD were mediated by SMuRFs, while the direct path between ln(HCC) and CAD was not statistically significant.

**Conclusion:**

The SMuRFs diabetes, hyperlipidemia, and hypertension mediate the association between ln(HCC) and CAD. Some association between ln(HCC) and CAD did not operate via the SMuRFs considered and might have a pathway through atherosclerosis or inflammation.

## Introduction

Coronary artery disease (CAD) is associated with the well-known standard modifiable risk factors (SMuRFs) diabetes, smoking, hypertension, and hyperlipidemia (1, 2). Consequently, prevention of CAD is focused on improving SMuRFs through modified lifestyle and by pharmacological treatment (3). However, about 15% of patients admitted with a primary ST-segment elevation myocardial infarction (STEMI) are SMuRF-less, i.e., nonsmokers without a diagnosis of diabetes, hypertension, or hyperlipidemia (2, 4–6). Notably, these SMuRF-less patients have increased mortality after STEMI compared to patients with SMuRFs (6). Thus, there is a need to find other nonstandard risk factors, which may also contribute to the development of atherosclerosis with subsequent CAD among SMuRF-less patients (4–6).

The glucocorticoid hormone cortisol has both the direct and indirect effects, which may increase the risk of CAD (7). For instance, patients suffering from glucocorticoid excess develop abdominal adiposity, dyslipidemia, hypertension, insulin resistance, and potentially type 2 diabetes (8). Moreover, taking prescribed glucocorticoids increase the risk of cardiovascular disease independent of underlying disease and common cardiovascular risk factors (9).

Cortisol has a considerable diurnal rhythmicity, which complicates sampling of this hormone (10). To overcome this difficulty, methods have been developed to measure cortisol levels in hair, which give a measure of the cumulative cortisol exposure in retrospect (11, 12). Notably, patients suffering from an acute MI (AMI) have increased levels of cortisol in hair prior weeks to the event compared with healthy controls (13) and patients treated for other diseases (14). High hair cortisol concentrations (HCCs) have also been shown among patients with the SMuRFs diabetes (15–17), hypertension (18), and smoking (15), as well as other cardiovascular risk factors including abdominal obesity (19), low high-density lipoprotein (HDL)-cholesterol levels (19), and a history of cardiovascular disease (17). Importantly, longitudinal data suggest that higher HCC is associated with an increased weight gain over 3 years (20). Thus, it may be suggested that increased circulating cortisol levels, either due to Cushing's syndrome (8), glucocorticoid treatment (9), or other more subtle derangements of the hypothalamic-pituitary-adrenal (HPA) axis reflected by increased HCC (20), is a cause rather than a consequence of obesity and SMuRFs.

Even though increased cortisol exposure seems to be a risk factor for CAD, it is not clear to what degree this is an indirect effect mediated by SMuRFs such as dyslipidemia, hypertension, and diabetes or a direct effect on the development of CAD (21). Therefore, we sought to use path analysis to test the direct and indirect association, mediated by SMuRFs, between HCC and CAD.

## Methods

### Study Design and Participants

**Figure 1:**
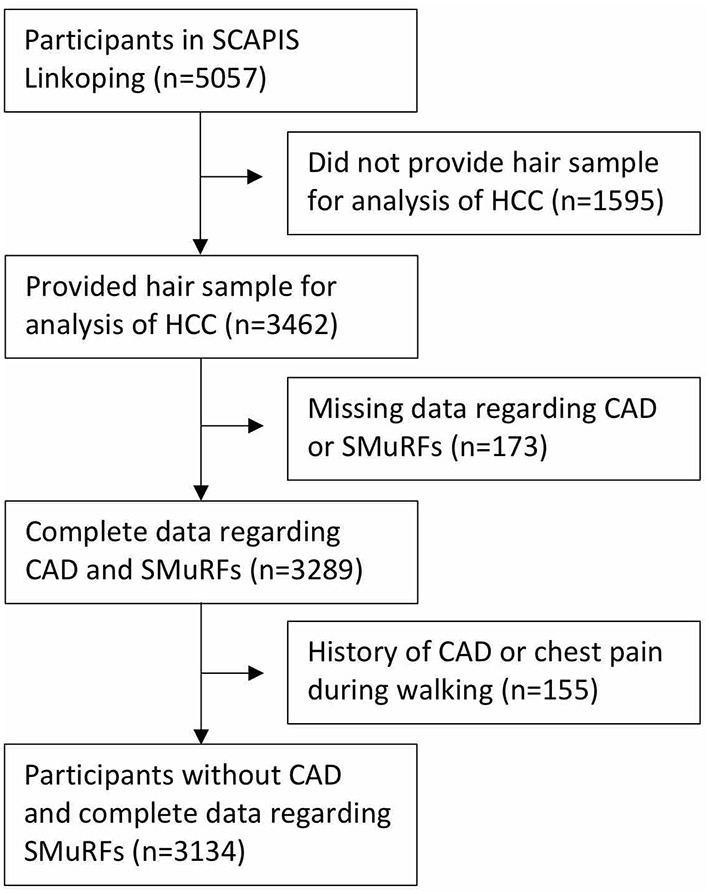
Flow diagram over included participants from the SCAPIS study. HCC, hair cortisol concentration; CAD, coronary artery disease; SMuRF, standard modifiable risk factor; SCAPIS, Swedish CArdioPulmonary BioImage Study.

This was a cross-sectional study including participants from two studies—the Stressheart study (13) and the Swedish CArdioPulmonary BioImage Study (SCAPIS) (22). A detailed description and main results from the Stressheart study have been published previously (13). The Stressheart study included 203 patients admitted with a STEMI or non-STEMI (NSTEMI) in the southeast healthcare region of Sweden during year 2016–2019. Patients with a hair length < 1 cm, not speaking Swedish language, and older than 65 years were excluded. The SCAPIS study was conducted during year 2015–2018 at six universities in Sweden. Data in this study were acquired from participants included at Linköping University situated in the same southeast hospital region as in which the Stressheart study was conducted. The SCAPIS study included men and women between 50 and 65 years of age randomly sampled from the population. In total, 5,057 participants were included at Linköping University of whom 3,462 participants provided a hair sample for cortisol analysis. Of these 3,462 participants, 155 participants reported a history of MI (*n* = 46), angina (*n* = 25), previous coronary artery bypass graft surgery or percutaneous coronary artery intervention (*n* = 40), and chest pain when walking on flat ground (*n* = 16) or uphill (*n* = 78), and were, therefore, excluded. Furthermore, another 173 participants who lacked information on history of CAD, chest pain, or smoking status were also excluded leaving 3,134 participants from the SCAPIS study (Figure 1). The Stressheart and the SCAPIS studies were approved by the Regional Ethical Review Boards (Dnr 2016-79-31, Dnr 2016-453-32, Dnr 2017-106-32, and Dnr 2010-228-31M). All the participants gave their written informed consent to participate.

### Definition of SMuRFs

Standard modifiable risk factors were defined as having a diagnosis of, or pharmacotherapy for, diabetes, hypertension, or hyperlipidemia, which have been used in previous studies on the role of SMuRFs in CAD (4–6). This was reported by the patient in the SCAPIS study and checked in the patient file in the Stressheart study. A total cholesterol ≥ 5.5 mmol/l and/or low-density lipoprotein (LDL)-cholesterol ≥ 3.5 mmol/l was also used to define participants with hyperlipidemia (4–6). Regular smoking of at least one cigarette/day during the last month was also considered as SMuRF (6). The blood pressure level and plasma glucose level were not included in the definition, since single measurements of these parameters were not considered enough to firmly diagnose participants as being hypertensive or having diabetes in either the SCAPIS or the Stressheart studies. In total, 2,141 participants in the SCAPIS study reported having at least one SMuRF. In the Stressheart study, 173 participants had at least one SMuRF at the time of admission with AMI whereas 30 participants were SMuRF-less at the time of admission (Table 1).

**Table 1:** Characteristics of included participants grouped according to coronary artery disease (CAD) status.

	**Coronary artery disease (*n* = 203)**	**No coronary artery disease** **(*n* = 3,134)**	**P-value**
Female	56 (28)	1,994 (64)	< 0.0001
Age	57.7 (6.5)	57.3 (4.4)	0.002
Educational level			< 0.0001
Elementary school	47 (23)	251 (8)	
Upper secondary school	113 (56)	1,472 (47)	
University or similar	43 (21)	1,406 (45)	
Born in Sweden	176 (87)	2,957 (94)	< 0.0001
STEMI	112 (57)	NA	
Previous MI	51 (26)	NA	
Heredity for MI	86 (51)	205 (7)	< 0.0001
Hyperlipidemia	56 (32)	255 (8)	< 0.0001
Hypertension	81 (40)	570 (18)	< 0.0001
Diabetes	31 (16)	115 (4)	< 0.0001
Active smoker	56 (28)	271 (9)	< 0.0001
SMuRFs			< 0.0001
0	54 (32)	2,210 (71)	
1	62 (36)	688 (22)	
2	30 (18)	186 (6)	
3	24 (14)	39 (1)	
4	1 (0.6)	8 (0.3)	
BMI (kg/m^2^)	27.8 (4.4)	26.8 (4.5)	< 0.0001
Waist circumference (cm)	100 (13)	91 (13)	< 0.0001
P-glucose (mmol/L)	7.2 (3.0)	5.6 (1.1)	< 0.0001
Total cholesterol (mmol/L)	5.1 (1.3)	5.5 (1.0)	< 0.0001
LDL-cholesterol (mmol/L)	3.0 (1.2)	3.3 (0.9)	< 0.0001
HDL-cholesterol (mmol/L)	1.19 (0.40)	1.70 (0.50)	< 0.0001
Triglycerides (mmol/L)	2.1 (1.3)	1.9 (0.7)	< 0.0001
Systolic blood pressure (mmHg)	125 (17)	132 (18)	< 0.0001
Diastolic blood pressure (mmHg)	79 (12)	83 (10)	< 0.0001
Hair cortisol concentration (pg/mg)	75.2 (167.1)	23.6 (35.0)	< 0.0001

*Categorical data is presented as n (%) and continuous data as mean (SD) except for the hair cortisol concentration, which is given as median [interquartile range (IQR)]. Prevalence of hyperlipidemia, hypertension, and diabetes were based on either a diagnosis or pharmacological treatment. p-value denotes the significance level of the difference between participants with and without coronary artery disease*.

### Outcomes

The primary outcome in this study was the direct and indirect association between HCC and CAD. A detailed description on the hair sampling and analysis of HCC has been published previously (13). At least 1 cm of hair, which represents the previous 4–6 weeks, was sampled from the posterior vertex of the scalp (23). The HCC was analyzed using a competitive radioimmunoassay (RIA), since it is suitable for small samples of hair. Hair samples from both the Stressheart and the SCAPIS studies were analyzed during the same time period (2016–2019) in the same laboratory under the same protocol. All the participants in both studies answered questionnaires with respect to educational level, ethnicity, current pharmacotherapy, present and previous diseases including angina, congestive heart failure, MI, stroke, previous coronary artery bypass grafting or percutaneous coronary intervention, diabetes, hyperlipidemia, and if any first-degree relatives suffered from MI. Smoking status was reported by the participant. Length, weight, waist circumference, and blood pressure on the day of discharge from the hospital in the Stressheart study were measured by a nurse. In the SCAPIS study, a fasting blood sample was drawn and plasma glucose, serum cholesterol, LDL-cholesterol, HDL-cholesterol, and triglycerides were analyzed according to clinical routine (22). In the Stressheart study, data on these biochemical measures were extracted from the Swedish Web-system for Enhancement and Development of Evidence-based care in Heart disease Evaluated According to Recommended Therapies (SWEDEHEART), which is a national register of patients treated in coronary care units (24).

### Statistical Analysis

All the data were checked for normal distribution. The HCC was not normally distributed and was, therefore, logarithmically transformed using the natural logarithm before inclusion in the statistical models [ln(HCC)]. This transformation also avoids the long tail of raw cortisol values having undue influence on the associations with CAD. All the continuous data were presented as mean with SD, except for HCC, which was presented as median (interquartile range) and discrete data as number (n) with proportion in percent.

Group comparisons were made between participants from the Stressheart and the SCAPIS studies (Table 1). Categorical measures were compared using the Pearson's chi-squared test, while quantitative measures were compared between the groups using the nonparametric Mann–Whitney *U* test.

Path analysis was used to test the direct and indirect association between ln(HCC) and CAD. The SMuRFs such as hypertension, hyperlipidemia, and diabetes were included as mediators, while smoking, gender, and educational level were included as confounders. Education was treated as an ordinal measure with elementary school as the reference. The confounders were included, since they were unevenly distributed between participants with and without CAD and are known risk factors for CAD (Table 1). Due to the highly parametrized nature of path models, some screening of potential SMuRF measures to be included was undertaken to avoid potential estimation problems. Collinearity was evaluated via the variance inflation factor (VIF), although no clear problems (VIF > 10) were identified. Since model parameter estimation did not converge, measures were omitted one at a time based on the largest VIF values and on a priori strength of evidence To be a potential source of indirect association, a variable needed to be associated with both the ln(HCC) and with CAD. This analysis is reported in Table 2 using linear regression with statistical inference via the nonparametric bootstrap for ln(HCC) and unconditional logistic regression for CAD. Only SMuRF measures that met both the criteria were included in path models.

**Table 2:** Screening of potential mediators to be included in the path analysis.

**Predictor**	**Ln(HCC)**		**Coronary artery disease**	
	**Beta**	**P-value**	**Odds ratio**	**P-value**
Hypertension	0.15 (0.05–0.24)	0.003	2.99 (2.22–4.01)	< 0.0001
Diabetes	0.41 (0.21–0.62)	< 0.0001	4.82 (3.15–7.37)	< 0.0001
Hyperlipidemia	0.30 (0.17–0.44)	< 0.0001	5.35 (3.80–7.54)	< 0.0001
Smoking	0.01 (−0.12–0.14)	0.86	4.11 (2.95–5.73)	< 0.0001
LDL-cholesterol	−0.02 (−0.06–0.03)	0.45	0.70 (0.59–0.82)	< 0.0001
HDL-cholesterol	−0.31 (−0.38–−0.23)	< 0.0001	0.049 (0.031–0.079)	< 0.0001
Triglycerides	0.14 (0.09–0.20)	< 0.0001	2.16 (1.89–2.47)	< 0.0001
Waist circumference	0.10 (0.07–0.13)	< 0.0001	1.047 (1.037–1.058)	< 0.0001
Female	−0.41 (−0.49–−0.33)	< 0.0001	0.22 (0.16–0.30)	< 0.0001
Educational level	−0.04 (−0.10–0.020)	0.17	0.40 (0.33–0.50)	< 0.0001

*The associations were tested using univariate linear regression when logarithmized hair cortisol concentration [ln(HCC)] was the outcome and univariate logistic regression when coronary artery disease was the outcome. Male and lowest level of education (elementary school) was used as reference for these predictors. Raw beta values and hazard ratios are presented with 95% CIs*.

The quantitative measures reflecting SMuRFs such as the level of diastolic and systolic blood pressure, fasting plasma glucose, serum triglycerides, HDL-cholesterol, LDL-cholesterol, total cholesterol, and waist circumference were omitted from the model reported in Table 3, despite meeting criteria for inclusion, due to their correlation with SMuRFs inducing multicollinearity. The estimated direct, indirect, and total path coefficients (b) are shown in Table 3, along with SE and *p*-value. The percentage of the association between ln(HCC) and CAD that is estimated to be indirect is calculated from indirect ÷ total × 100. Models were fitted in MPlus version 8 using maximum likelihood and with inference derived via the nonparametric bootstrap with 2,000 bootstrap samples. Components of the paths model were estimated using link functions appropriate to the dependent variable measurement scale, identity link for quantitative outcomes, and logit link for binary outcomes. It is noted that due to the cross-sectional design of this study, causal inference cannot be made from these models and purpose of path modeling is to understand the extent to which the associations of interest might be operating via other factors. Any causal modeling would require a future longitudinal study.

**Table 3:** Direct and indirect associations between ln(HCC) and CAD.

	**b**	**SE**	**z**	**P-value**
Total	0.349	0.026	13.464	< 0.0001
Total indirect	0.280	0.062	4.508	< 0.0001
*Specific indirect*				
Hypertension	0.058	0.015	3.812	< 0.0001
Diabetes	0.103	0.031	3.373	0.001
Hyperlipidemia	0.119	0.026	4.505	< 0.0001
Total direct	0.069	0.064	1.070	0.285

*Estimates (b) are path coefficients expressing the association between the ln(HCC) as predictor and CAD as outcome either directly or indirectly via several standard modifiable risk factor (SMuRF) measures. The total direct + total indirect = Total*.

A *p* < 0.05 was considered significant in all the analyses. Statistical analyses shown in Tables 1, 2 were performed in SPSS version 25 and Stata version 17 for bootstrapping, while the path model shown in Table 3 were estimated using MPlus version 8.

## Results

### Age, Gender, Ethnicity, and Educational Level

The mean age was 57 years among participants with and without CAD. A greater proportion of participants with CAD were males (72 vs. 36%, *p* < 0.0001). The educational level was lower among those with CAD and although most participants were born in Sweden, a larger proportion of participants with CAD had another ethnicity (Table 1).

### Hair Cortisol Concentrations, Cardiovascular Risk Factors, and Anthropometric Measurements

**Figure 2:**
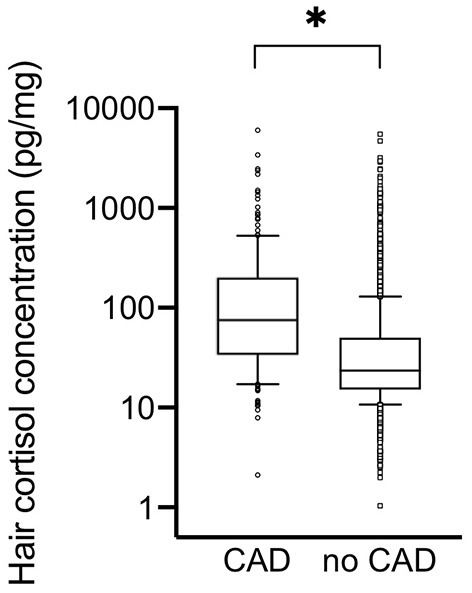
Hair cortisol concentration among participants with or without coronary artery disease (CAD). The hair cortisol concentration given on a logarithmic scale. The line represents the median, box 25th to 75th percentile, and whiskers 10th to 90th percentile. *p < 0.0001 for difference between the groups.

Participant characteristics are given in Table 1. Heredity for MI was considerably higher among participants with CAD. Participants with CAD also had a higher body mass index (BMI) and larger waist circumference than participants without CAD. The levels of fasting plasma glucose and triglycerides were higher among participants with CAD, while the systolic and diastolic blood pressure levels as well as total cholesterol, HDL-cholesterol, and LDL-cholesterol levels were lower. The HCC was three times as high (*p* < 0.0001) among participants with CAD compared to those without CAD (Figure 2).

### Standard Modifiable Risk Factors

A total of 32% of participants with CAD had no SMuRFs, while 71% of those participants without CAD were SMuRF-less (Table 1). Hypertension was the most common SMuRF independently of CAD status (Table 1). Participants with CAD had a significantly higher number of SMuRFs compared to participants without CAD (Table 1).

### Associations Between HCC, Cardiovascular Risk Factors, and CAD

As shown in Table 2, higher ln(HCC) was associated with a diagnosis of, or treatment for, hypertension, diabetes, and hyperlipidemia, but not active smoking. Furthermore, a higher ln(HCC) was also associated with lower HDL-cholesterol, higher triglyceride levels, and a greater waist circumference. Being female was associated with lower ln(HCC). Ln(HCC) was associated with CAD [hazard ratio (HR) 1.86 (95% CI 1.68–2.06), *p* < 0.0001]. An increased risk of CAD was present among participants with hypertension, diabetes, hyperlipidemia, and active smoking. Lower HDL-cholesterol and higher triglyceride levels were also associated with CAD as well as a large waist circumference, low educational level, and being male were also associated with CAD. Notably, higher LDL-cholesterol levels were associated with lower risk of CAD.

### Direct and Indirect Associations Between HCC and CAD

**Figure 3:**
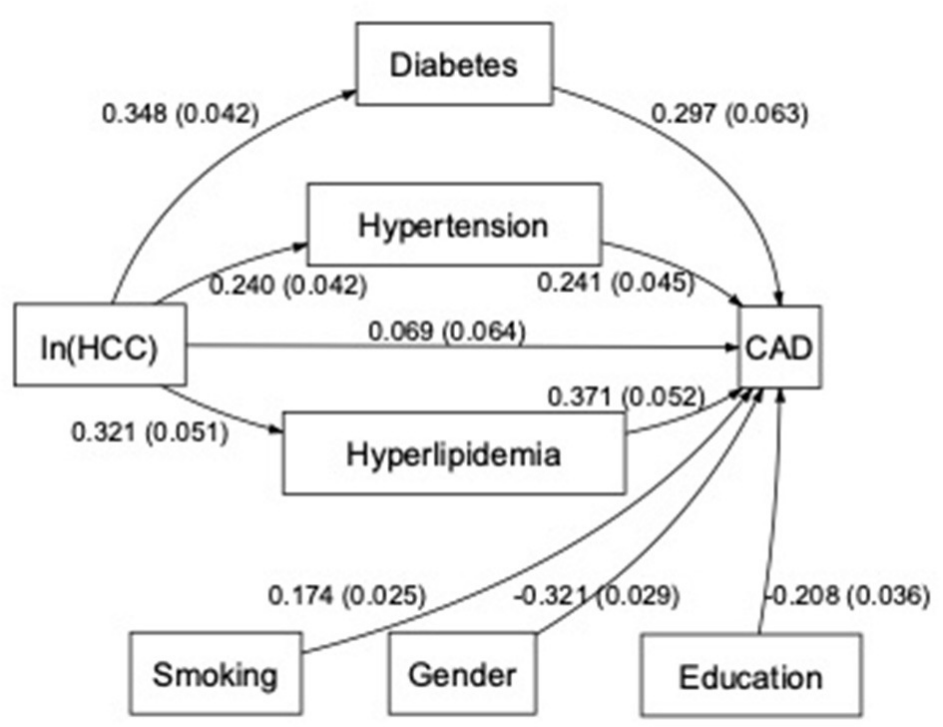
Path diagram over the associations between the HCC, CAD, and standard modifiable cardiovascular risk factors. Boxes represent included variables in the path model and arrows the estimated associations (with the SE in parentheses) between variables in the path model. The arrow from logarithmized HCC [ln(HCC)] to CAD represents the direct association and the others represent the indirect associations. Smoking, gender with male as reference, and education with elementary school as reference were included as confounders in the model.

The path model, including the SMuRFs such as diabetes, hypertension, and hyperlipidemia as mediators and smoking, gender, and educational level as confounders, resulted in 80% (0.280/0.349) indirect association between ln(HCC) and CAD (Figure 3). Although the direct path between ln(HCC) and CAD was not statistically significant, it was estimated that 20% of the association between ln(HCC) and CAD was direct and, therefore, did not operate via the SMuRFs considered. Specifically, hyperlipidemia (*b* = 0.119), closely followed by diabetes (*b* = 0.103) was the strongest indirect paths between HCC and CAD (Figure 3).

## Discussion

This study shows that HCC was considerably higher among participants with CAD compared with a healthy population-based sample without a history or symptoms of CAD. The ln(HCC) was also strongly associated with both SMuRFs and other cardiovascular risk factors such as abdominal obesity, high triglyceride levels, and low HDL-cholesterol levels. Notably, about 80% of the association between ln(HCC) and CAD were indirect, suggesting that these cardiovascular risk factors mediated the association between ln(HCC) and CAD.

The aim of this study was to elucidate upon long-term cortisol exposure as a risk factor for CAD. This was based upon the fact that the SMuRFs such as hypertension, hyperlipidemia, diabetes, and smoking, that are the major contributors of CAD, do not explain development of CAD among all the patients (1). About 15% of patients with a STEMI are SMuRF-less and other risk factors, in addition to SMuRFs, have been sought for (4–6). Since both the iatrogenic and pathological states with cortisol excess increase cardiovascular risk, excessive long-term cortisol exposure is a plausible cardiovascular risk factor in addition to SMuRFs (7, 9, 25). Our results contradict this hypothesis and suggests that the relationship between ln(HCC) and CAD is mainly indirect and mediated by SMuRFs, rather than direct. At first sight, this might seem contradictive to previous studies showing that patients taking prescribed glucocorticoids have increased cardiovascular risk independent of other cardiovascular risk factors (9) and that patients admitted with an AMI have higher HCC prior to the event compared with healthy controls, independent of other cardiovascular risk factors (13, 14). However, estimation of indirect pathways does not equate to an analysis of confounding, but rather estimates how much of the association might be via the SMuRFs considered. Thus, the associations between glucocorticoids and CAD, shown previously that have been independent of cardiovascular risk factors in regression models, do not rule out that the cardiovascular risk factors mediated these associations (9, 13, 14, 26). The clinical implication of this finding is that the increased cortisol levels observed among patients with CAD seems to be more related to the SMuRFs rather than CAD. However, this does not necessarily mean that the role of increased cortisol levels in CAD is unimportant, since it may still increase the risk of SMuRFs. Future longitudinal studies could provide further insight of cause-and-effect in this relationship.

As expected, participants with CAD were mainly males, had lower education, more heredity for MI, a higher number of SMuRFs, larger waist circumference, higher triglyceride, and lower HDL-cholesterol levels as well as higher fasting plasma blood glucose compared to participants without CAD. The total cholesterol level, LDL-cholesterol level, and blood pressure were lower due to a higher rate of pharmacotherapy for hypertension and hyperlipidemia. When screening for potential mediators, several of these quantitative measures were associated with both the ln(HCC) and CAD, qualifying for inclusion in the path model. However, due to multicollinearity, the quantitative measures could not be included as mediators in the final path analysis, but gender, smoking, and educational level were included as confounders.

The strong indirect association between ln(HCC) and CAD does not necessarily equate to a causative effect. A longitudinal or experimental study would be needed to draw such conclusion (26). However, one suggested mechanism may be that glucocorticoids induce insulin resistance and increased blood glucose levels, which are supported by strong associations between increased HCC and the metabolic syndrome and type 2 diabetes (16, 17, 19, 27). Furthermore, several studies imply that individuals with abdominal obesity, which is considered a main cause for insulin resistance in the metabolic syndrome (28), have increased activity in the HPA axis and, therefore, increased cortisol output. Whether the increased cortisol output is a cause or consequence of the abdominal obesity is, however, still debated (29–33).

Data in this article were gathered from two different cohorts. Importantly though, participants in both the cohorts were living in the same geographical region, hair was sampled, and cortisol levels analyzed under the same protocol in the same laboratory during the same period, minimizing the risk for systematic errors in this process. Among participants with CAD, the hair sample was taken during the hospital admission for AMI. Importantly, the first part of the hair strand, reflecting the last week, is located underneath the scalp, and, therefore, not associated with the acute stress from the AMI (34). However, it cannot be excluded that some participants with CAD had angina and chest pain the weeks before the AMI, which could potentially increase stress and, thereby, the HCC. But according to previous studies, the relationship between subjective stress and HCC is limited and only a small proportion of patients with AMI seems to seek medical care due to prodromal symptoms before the actual event (35, 36). Therefore, we consider it unlikely that the increased HCC among patients with CAD were caused by acute stress related to the subsequent AMI, rather than chronically elevated HCC levels. We also note that an analysis of indirect association paths does not literally equate to an analysis of mediation, which has a causal interpretation and, therefore, requires a longitudinal study design.

The diagnosis of CAD was set by the cardiologist at the cardiology department and can, therefore, be considered of high quality. Participants from the SCAPIS study (without CAD) answered a questionnaire including questions on previous MI, angina pectoris, percutaneous coronary intervention, and coronary artery bypass grafting. Furthermore, they were asked about symptoms of chest pain when walking. All the participants answering yes to one of these questions were excluded. Despite being asked explicitly, it cannot be fully granted that none of these participants had been diagnosed with CAD previously. Furthermore, they could have atherosclerotic plaques in coronary arteries despite being asymptomatic. Thus, future studies using noninvasive methods such as coronary computational tomography to examine the prevalence of atherosclerotic coronary artery disease, irrespective of a diagnosis or symptoms of CAD, in relation to cortisol levels could be of great interest. Notably, a recent study suggests that angiographically diagnosed CAD is associated with increased HCC, independent of other cardiovascular risk factors (37). Furthermore, adding information on psychosocial stress would also be interesting, since it is a risk factor for CAD (38) and has been suggested to be linked to increased HCC as well (39).

In conclusion, the association between ln(HCC) and CAD is mainly indirect, mediated by SMuRFs. This suggests that individuals with chronically elevated cortisol levels are more likely to also have cardiovascular risk factors and, thereby, increased cardiovascular risk. However, around 20% of the association between ln(HCC) and CAD is not explained by SMuRFs. Therefore, future studies might also focus on the relation between HCC and atherosclerosis or inflammation (20). Finally, even though studies suggest that increased cortisol levels cause obesity and metabolic dysregulation (8, 9, 20), future longitudinal studies could investigate the cause-and-effect relationship between increased HCC, SMuRFs, and CAD even further.

## Previous Presentations

Part of this data focusing on a comparison of the hair cortisol concentration among participants in the Stressheart study and healthy controls have been published previously in Faresjo et al. (13). This article includes more participants with coronary artery disease and addresses the direct and indirect associations between hair cortisol concentrations and coronary artery disease by using path analysis, which was not addressed in the previous article.

## Data Availability Statement

The raw data supporting the conclusions of this article will be made available upon request to the authors, if it is in accordance with laws and regulations.

## Ethics Statement

The studies involving human participants were reviewed and approved by Region Ethical Review Board of Umeå and Linköping. The patients/participants provided their written informed consent to participate in this study.

## Author Contributions

AS: planning and data collection, data analysis, and lead author of manuscript. SS: planning and data collection, data analysis, and co-author of manuscript. ET: development of analysis method and analysis of hair samples, data analysis, and co-author of manuscript. ÅO: design, planning and data collection, and co-author of manuscript. MJ: statistical analysis and co-author of manuscript. TF: principal investigator, design, planning and data collection, data analysis, and co-author of manuscript. All authors contributed to the article and approved the submitted version.

## Funding

This study was funded by the Swedish AFA Insurance, Stockholm (Grant number Dnr: 160340).

## Conflict of Interest

The authors declare that the research was conducted in the absence of any commercial or financial relationships that could be construed as a potential conflict of interest.

## Publisher's Note

All claims expressed in this article are solely those of the authors and do not necessarily represent those of their affiliated organizations, or those of the publisher, the editors and the reviewers. Any product that may be evaluated in this article, or claim that may be made by its manufacturer, is not guaranteed or endorsed by the publisher.

## References

[1] Yusuf S Hawken S Ounpuu S Dans T Avezum A Lanas F. Effect of potentially modifiable risk factors associated with myocardial infarction in 52 countries (the INTERHEART study): case-control study. Lancet. (2004) 364:937–52. 10.1016/S0140-6736(04)17018-9 15364185

[2] Khot UN Khot MB Bajzer CT Sapp SK Ohman EM Brener SJ. Prevalence of conventional risk factors in patients with coronary heart disease. JAMA. (2003) 290:898–904. 10.1001/jama.290.7.898 12928466

[3] Knuuti J Wijns W Saraste A Capodanno D Barbato E Funck-Brentano C. 2019 ESC Guidelines for the diagnosis and management of chronic coronary syndromes. Eur Heart J. (2020) 41:407–77. 10.1093/eurheartj/ehz425 31504439

[4] Vernon ST Coffey S Bhindi R Soo Hoo SY Nelson GI Ward MR. Increasing proportion of ST elevation myocardial infarction patients with coronary atherosclerosis poorly explained by standard modifiable risk factors. Eur J Prev Cardiol. (2017) 24:1824–30. 10.1177/2047487317720287 28703626

[5] Vernon ST Coffey S D'Souza M Chow CK Kilian J Hyun K. ST-Segment-elevation myocardial infarction (STEMI) patients without standard modifiable cardiovascular risk factors-how common are they, and what are their outcomes? J Am Heart Assoc. (2019) 8:e013296. 10.1161/JAHA.119.013296 31672080 PMC6898813

[6] Figtree GA Vernon ST Hadziosmanovic N Sundström J Alfredsson J Arnott C. Mortality in STEMI patients without standard modifiable risk factors: a sex-disaggregated analysis of SWEDEHEART registry data. Lancet. (2021) 397:1085–94. 10.1016/S0140-6736(21)00272-5 33711294

[7] Neary NM Booker OJ Abel BS Matta JR Muldoon N Sinaii N. Hypercortisolism is associated with increased coronary arterial atherosclerosis: analysis of noninvasive coronary angiography using multidetector computerized tomography. J Clin Endocrinol Metab. (2013) 98:2045–52. 10.1210/jc.2012-3754 23559084 PMC3644598

[8] Chanson P Salenave S. Metabolic syndrome in Cushing's syndrome. Neuroendocrinology. (2010) 92 Suppl 1:96–101. 10.1159/000314272 20829627

[9] Wei L MacDonald TM Walker BR. Taking glucocorticoids by prescription is associated with subsequent cardiovascular disease. Ann Intern Med. (2004) 141:764–70. 10.7326/0003-4819-141-10-200411160-00007 15545676

[10] Lightman SL. The neuroendocrinology of stress: a never ending story. J Neuroendocrinol. (2008) 20:880–4. 10.1111/j.1365-2826.2008.01711.x 18601712

[11] Short SJ Stalder T Marceau K Entringer S Moog NK Shirtcliff EA. Correspondence between hair cortisol concentrations and 30-day integrated daily salivary and weekly urinary cortisol measures. Psychoneuroendocrinology. (2016) 71:12–8. 10.1016/j.psyneuen.2016.05.007 27235635 PMC4955743

[12] Karlen J Ludvigsson J Frostell A Theodorsson E Faresjo T. Cortisol in hair measured in young adults - a biomarker of major life stressors? BMC Clin Pathol. (2011) 11:12. 10.1186/1472-6890-11-12 22026917 PMC3217842

[13] Faresjo T Stromberg S Jones M Stomby A Karlsson JE Ostgren CJ. Elevated levels of cortisol in hair precede acute myocardial infarction. Sci Rep. (2020) 10:22456. 10.1038/s41598-020-80559-9 33384452 PMC7775435

[14] Pereg D Gow R Mosseri M Lishner M Rieder M Van Uum S. Hair cortisol and the risk for acute myocardial infarction in adult men. Stress. (2011) 14:73–81. 10.3109/10253890.2010.511352 20812871

[15] Feller S Vigl M Bergmann MM Boeing H Kirschbaum C Stalder T. Predictors of hair cortisol concentrations in older adults. Psychoneuroendocrinology. (2014) 39:132–40. 10.1016/j.psyneuen.2013.10.007 24275012

[16] Lehrer HM Dubois SK Maslowsky J Laudenslager ML Steinhardt MA. Hair cortisol concentration and glycated hemoglobin in African American adults. Psychoneuroendocrinology. (2016) 72:212–8. 10.1016/j.psyneuen.2016.06.018 27500952

[17] Manenschijn L Schaap L van Schoor NM van der Pas S Peeters GM Lips P. High long-term cortisol levels, measured in scalp hair, are associated with a history of cardiovascular disease. J Clin Endocrinol Metab. (2013) 98:2078–83. 10.1210/jc.2012-3663 23596141

[18] Stalder T Kirschbaum C. Analysis of cortisol in hair–state of the art and future directions. Brain Behav Immun. (2012) 26:1019–29. 10.1016/j.bbi.2012.02.002 22366690

[19] Stalder T Kirschbaum C Alexander N Bornstein SR Gao W Miller R. Cortisol in hair and the metabolic syndrome. J Clin Endocrinol Metab. (2013) 98:2573–80. 10.1210/jc.2013-1056 23585660

[20] van der Valk ES van der Voorn B Iyer AM Mohseni M Leenen PJM Dik WA. Hair cortisol, obesity and the immune system: Results from a 3 year longitudinal study. Psychoneuroendocrinology. (2021) 134:105422. 10.1016/j.psyneuen.2021.105422 34666286

[21] Kipari T Hadoke PW Iqbal J Man TY Miller E Coutinho AE. 11beta-hydroxysteroid dehydrogenase type 1 deficiency in bone marrow-derived cells reduces atherosclerosis. FASEB J. (2013) 27:1519–31. 10.1096/fj.12-219105 23303209 PMC3606528

[22] Bergstrom G Berglund G Blomberg A Brandberg J Engstrom G Engvall J. The Swedish CArdioPulmonary BioImage Study: objectives and design. J Intern Med. (2015) 278:645–59. 10.1111/joim.12384 26096600 PMC4744991

[23] Wennig R. Potential problems with the interpretation of hair analysis results. Forensic Sci Int. (2000) 107:5–12. 10.1016/S0379-0738(99)00146-2 10689559

[24] Jernberg T Attebring MF Hambraeus K Ivert T James S Jeppsson A. The Swedish Web-system for enhancement and development of evidence-based care in heart disease evaluated according to recommended therapies (SWEDEHEART). Heart. (2010) 96:1617–21. 10.1136/hrt.2010.198804 20801780

[25] Fardet L Petersen I Nazareth I. Risk of cardiovascular events in people prescribed glucocorticoids with iatrogenic Cushing's syndrome: cohort study. BMJ: British Medical Journal. (2012) 345:e4928. 10.1136/bmj.e4928 22846415 PMC3408386

[26] MacKinnon DP Fairchild AJ Fritz MS. Mediation analysis. Annu Rev Psychol. (2007) 58:593–614. 10.1146/annurev.psych.58.110405.085542 16968208 PMC2819368

[27] Staufenbiel SM Penninx BW de Rijke YB van den Akker EL van Rossum EF. Determinants of hair cortisol and hair cortisone concentrations in adults. Psychoneuroendocrinology. (2015) 60:182–94. 10.1016/j.psyneuen.2015.06.011 26176863

[28] Alberti KG Eckel RH Grundy SM Zimmet PZ Cleeman JI Donato KA. Harmonizing the metabolic syndrome: a joint interim statement of the International Diabetes Federation Task Force on Epidemiology and Prevention; National Heart, Lung, and Blood Institute; American Heart Association; World Heart Federation; International Atherosclerosis Society; and International Association for the Study of Obesity. Circulation. (2009) 120:1640–5. 10.1161/CIRCULATIONAHA.109.192644 19805654

[29] Bujalska IJ Kumar S Stewart PM. Does central obesity reflect Cushing's disease of the omentum? Lancet. (1997) 349:1210–3. 10.1016/S0140-6736(96)11222-8 9130942

[30] Stimson RH Andersson J Andrew R Redhead DN Karpe F Hayes PC. Cortisol release from adipose tissue by 11beta-hydroxysteroid dehydrogenase type 1 in humans. Diabetes. (2009) 58:46–53. 10.2337/db08-0969 18852329 PMC2606892

[31] Marin P Darin N Amemiya T Andersson B Jern S Bjorntorp P. Cortisol secretion in relation to body fat distribution in obese premenopausal women. Metabolism. (1992) 41:882–6. 10.1016/0026-0495(92)90171-6 1640867

[32] Pasquali R Cantobelli S Casimirri F Capelli M Bortoluzzi L Flamia R. The hypothalamic-pituitary-adrenal axis in obese women with different patterns of body fat distribution. J Clin Endocrinol Metab. (1993) 77:341–6. 10.1210/jcem.77.2.8393881 8393881

[33] Purnell JQ Kahn SE Samuels MH Brandon D Loriaux DL Brunzell JD. Enhanced cortisol production rates, free cortisol, and 11beta-HSD-1 expression correlate with visceral fat and insulin resistance in men: effect of weight loss. Am J Physiol Endocrinol Metab. (2009) 296:E351–7. 10.1152/ajpendo.90769.2008 19050176 PMC2645022

[34] Pragst F Balikova MA. State of the art in hair analysis for detection of drug and alcohol abuse. Clin Chim Acta. (2006) 370:17–49. 10.1016/j.cca.2006.02.019 16624267

[35] Rietschel L Streit F Zhu G McAloney K Frank J Couvy-Duchesne B. Hair cortisol in twins: heritability and genetic overlap with psychological variables and stress-system genes. Sci Rep. (2017) 7:15351. 10.1038/s41598-017-11852-3 29127340 PMC5703444

[36] Graham MM Westerhout CM Kaul P Norris CM Armstrong PW. Sex differences in patients seeking medical attention for prodromal symptoms before an acute coronary event. Am Heart J. (2008) 156:1210–6 e1. 10.1016/j.ahj.2008.07.016 19033022

[37] Nafisa A Wattoo FH Qayyum M Gulfraz M. The association between chronic stress, hair cortisol, and angiographically documented coronary atherosclerosis, a case-control study. Stress. (2021) 1–8. 10.1080/10253890.2021.1985994 34633899

[38] Rosengren A Hawken S Ounpuu S Sliwa K Zubaid M Almahmeed WA. Association of psychosocial risk factors with risk of acute myocardial infarction in 11119 cases and 13648 controls from 52 countries (the INTERHEART study): case-control study. Lancet. (2004) 364:953–62. 10.1016/S0140-6736(04)17019-0 15364186

[39] Fischer S Duncko R Hatch SL Papadopoulos A Goodwin L Frissa S. Sociodemographic, lifestyle, and psychosocial determinants of hair cortisol in a South London community sample. Psychoneuroendocrinology. (2017) 76:144–53. 10.1016/j.psyneuen.2016.11.011 27923182

